# Mapping *Schistosoma mansoni* endemicity in Rwanda: a critical assessment of geographical disparities arising from circulating cathodic antigen versus Kato-Katz diagnostics

**DOI:** 10.1371/journal.pntd.0007723

**Published:** 2019-09-30

**Authors:** Nicholas J. Clark, Irenee Umulisa, Eugene Ruberanziza, Kei Owada, Daniel G. Colley, Giuseppina Ortu, Carl H. Campbell, Emmanuel Ruzindana, Warren Lancaster, Jean Bosco Mbonigaba, Aimable Mbituyumuremyi, Alan Fenwick, Ricardo J. Soares Magalhaes, Innocent Turate

**Affiliations:** 1 UQ Spatial Epidemiology Laboratory, School of Veterinary Science, The University of Queensland, Queensland, Australia; 2 Children Health and Environment Program, Child Health Research Centre, The University of Queensland, Queensland, Australia; 3 Neglected Tropical Diseases and Other Parasitic Diseases Unit, Malaria and Other Parasitic Diseases Division, Rwanda Biomedical Center, Ministry of Health, Kigali, Rwanda; 4 Center for Tropical and Emerging Global Diseases, Department of Microbiology, University of Georgia, Georgia, United States of America; 5 Schistosomiasis Control Initiative (SCI), Department of Infectious Diseases Epidemiology, Imperial College, London, United Kingdom; 6 Microbiology Unit, National Reference Laboratory (NRL) Division, Rwanda Biomedical Center, Ministry of Health, Kigali, Rwanda; 7 The END Fund, New York, New York, United States of America; 8 Malaria and Other Parasitic Diseases Division, Rwanda Biomedical Center, Ministry of Health, Kigali, Rwanda; 9 Institute of HIV/AIDS, Disease Prevention and Control (IHDPC), Rwanda Biomedical Center, Ministry of Health, Kigali, Rwanda; London School of Hygiene and Tropical Medicine, UNITED KINGDOM

## Abstract

**Background:**

Schistosomiasis is a neglected tropical disease caused by *Schistosoma* parasites. Intervention relies on identifying high-risk regions, yet rapid *Schistosoma* diagnostics (Kato-Katz stool assays (KK) and circulating cathodic antigen urine assays (CCA)) yield different prevalence estimates. We mapped *S*. *mansoni* prevalence and delineated at-risk regions using a survey of schoolchildren in Rwanda, where *S. mansoni* is an endemic parasite. We asked if different diagnostics resulted in disparities in projected infection risk.

**Methods:**

Infection data was obtained from a 2014 Rwandan school-based survey that used KK and CCA diagnostics. Across 386 schools screened by CCA (N = 19,217). To allow for uncertainty when interpreting ambiguous CCA *trace* readings, which accounted for 28.8% of total test results, we generated two presence-absence datasets: *CCA trace as positive* and *CCA trace as negative*. Samples (N = 9,175) from 185 schools were also screened by KK. We included land surface temperature (LST) and the Normalized Difference Vegetation and Normalized Difference Water Indices (NDVI, NDWI) as predictors in geostatistical regressions.

**Findings:**

Across 8,647 children tested by both methods, prevalence was 35.93% for *CCA trace as positive*, 7.21% for *CCA trace as negative* and 1.95% for KK. LST was identified as a risk factor using KK, whereas NDVI was a risk factor for CCA models. Models predicted high endemicity in Northern and Western regions of Rwanda, though the *CCA trace as positive* model identified additional high-risk areas that were overlooked by the other methods. Estimates of current burden for children at highest risk (boys aged 5–9 years) varied by an order of magnitude, with 671,856 boys projected to be infected by *CCA trace as positive* and only 60,453 projected by *CCA trace as negative* results.

**Conclusions:**

Our findings show that people in Rwanda’s Northern, Western and capital regions are at high risk of *S*. *mansoni* infection. However, variation in identification of environmental risk factors and delineation of at-risk regions using different diagnostics likely provides confusing messages to disease intervention managers. Further research and statistical analyses, such as latent class analysis, can be used to improve CCA result classification and assess its use in guiding treatment regimes.

## Author summary

Schistosomiasis control efforts rely on rapid diagnostic tests to generate maps of disease prevalence. This is so that high-risk areas can be identified and provided with treatments. A range of tests are available, yet it is unclear how their differing results can inform disease mitigation. This study compared the results of diagnostic methods for uncovering areas at risk of *Schistosoma mansoni* infection in Rwanda. A national survey of Rwandan schoolchildren used two detection methods: a test detecting a schistosome antigen in urine (CCA) and a test to detect parasite eggs in stool (KK). We divided the CCA test into two datasets, as there is large uncertainty when interpreting its reading. We found that these three datasets all tell different stories about the distribution of *S*. *mansoni* in Rwanda. While all tests found similar high-risk areas where prevalence reaches 50%, such as Northern and Western regions, their overall messages are mixed. Some urine tests find areas that are high-risk, while stool tests in the same area may consider it low-risk. This makes it difficult to design budgets for treatment programs, as it is unclear where resources should go. More research and analyses are needed to determine how these tests can be used as tools for schistosomiasis risk mapping. Furthermore, the interpretations of mapping outcomes will also likely rest on national decisions regarding programmatic goals.

## Introduction

Schistosomiasis, caused by parasitic trematode species of the genus *Schistosoma*, is an infectious disease affecting people throughout the world’s tropical and sub-tropical regions [1, 2]. Chronic infections can lead to impaired growth and development in children, making schistosomiasis one of the world’s most important neglected tropical diseases [3]. Schistosomiasis is often linked to absence of access to latrines and / or clean water, which can lead to the use of freshwater bodies that can become contaminated with parasite eggs when infected people urinate or defecate while bathing, washing and swimming. Freshwater snails, often present in these water bodies, act as intermediate hosts for the parasites. Following infection of appropriate snail hosts, asexual replication occurs followed by the development of the infectious stages of the parasite which can be released into the water and infect people. Consequently, infection-related morbidity is especially common in poor agricultural areas that rely on unsanitized freshwater [4, 5]. Globally, it is estimated that more than 779 million people live in areas with high risk of human *Schistosoma* transmission [6]. Infection risk is particularly high in sub-Saharan Africa, where up to 90% of the world’s infections occur [6, 7]. Burdens in this region are enormous, matching or exceeding those related to malaria and HIV/AIDS [3]. An estimated 300,000 people die from schistosomiasis in Africa each year [8].

Mass delivery of anthelminthic treatment can reduce *Schistosoma* prevalence and facilitate major improvements to public health outcomes [9]. Mass administration of praziquantel can be cost effective, especially in areas with relatively high prevalence of infection and high transmission intensity [7, 10]. Yet burdens are so great that mass drug administration remains unaffordable for most low-income endemic countries [9, 11]. Steps have been taken by Merck KGaA, the United States Agency for International Development, the Bill and Melinda Gates Foundation, the British Department of International Development and the Global Network for Neglected Tropical Diseases to increase delivery of doses to sub-Saharan African countries [12].

Designing effective drug delivery programmes to reduce *Schistosoma* transmission is complicated by inadequate understanding of regional burdens and risk factors [13, 14]. The geographical distributions and transmission rates of *Schistosoma* parasites are poorly studied in many endemic regions but are broadly known to be driven by environmental heterogeneity [15–17]. Climate or environmental variables that influence soil moisture and composition can reflect variability in molluscan host distributions and parasite larval survival rates [18–20]. Data-driven approaches to identify environmental correlates of infection risk in understudied endemic regions are imperative to design mitigation procedures.

In addition to climatic and environmental heterogeneity in infection risk, uncertainty surrounding regional infection prevalence is a barrier to treatment design. The World Health Organization (WHO) recommends that treatment for schistosomiasis should be provided to areas of high endemism in efforts to reduce morbidity. Such treatment is important not only for morbidity control but also for current pushes to eliminate schistosomiasis where feasible [21]. Developing treatment regimens to achieve these goals relies on gathering accurate estimates of infection prevalence. Moreover, with targets for 2020–2030 now under public consultation [21], critical assessments of inferences resulting from different diagnostic tests are needed. This is particularly true considering that different diagnostics may be chosen depending on whether a country’s specified goal is elimination as a public health problem (morbidity control) or interruption of transmission. However, gathering estimates of prevalence is challenging, particularly since symptoms of intestinal schistosomiasis are incredibly variable and can include headache, fever, rash, anaemia, bloody diarrhoea and abdominal pain, hepatosplenomegaly, blood in urine, burning sensation during urination, fibrosis of the bladder, and specifically in females, genital lesions which may lead to irreversible consequences, including infertility [2, 22]. Use of rapid diagnostic tests is therefore recommended for generating estimates of prevalence. For intestinal schistosomiasis, detection of eggs in the faeces (primarily using the Kato-Katz (KK) method, which involves microscopic examination of faecal smears [23]) or of circulating cathodic antigen (CCA) in urine are the primary methods of choice for diagnosis of infections [24, 25]. However, the two methods can return different prevalence estimates, with CCA generally yielding higher sensitivity [26, 27]. The KK method may show particularly low sensitivity in low to moderate endemic areas, especially when there are many low-intensity infections [28].

Without an accurate understanding of current burdens and environmental correlates, identifying areas in need of treatment remains difficult. Geostatistical models that use up-to-date infection data and account for environmental risk factors are useful tools for producing evidence-based projections of populations at risk of *Schistosoma* infection [29, 30]. Modelling assessments should ideally be formulated following adequate scrutiny of assessment of the performances and inferences that are provided by different available diagnostic methods.

Rwanda is a landlocked central African country bordered by Tanzania to the East, Uganda to the North, the Democratic Republic of the Congo (DRC) to the West, and Burundi to the South. With a land area of 26,338 square kilometres and a population of over 10.7 million, Rwanda is one of the most densely populated countries in Africa [31]. The economy is mostly agriculture-based and the average life expectancy is 63 years [32]. Rwanda sustained widespread public health disruption as a result of political and social unrest throughout the 1990’s [33]. Health workforce training and public health outcomes have since improved, however, infectious disease monitoring and control still present major challenges [34–36]. Previous surveys suggest that *Schistosoma mansoni* is highly endemic and hence a parasite of high public health importance in Rwanda [34, 37]. While some evidence suggests intestinal schistosomiasis reaches 60% prevalence or higher in certain areas, research on infection rates is limited to small-scale regional studies [1, 38–40]. Consequently, we have a poor understanding of risk factors for schistosomiasis in Rwanda.

This study aims to understand geographical variation in *S*. *mansoni* infection risk in Rwanda and to provide new insights into uncertainties in treatment pathways that can arise from variation in chosen on-the-ground diagnostic procedures. To accomplish these aims, we outline two key objectives. First, we apply geostatistical models to data from a national school-based survey in Rwanda to identify *S*. *mansoni* risk factors, map infection prevalence and delineate endemic clusters of high risk. Second, we compare geographical patterns in projected infection risk arising from the use of the CCA and KK methods. We hypothesize that infection risk will be spatially clustered within the country but that geographical disparities in the size and intensity of these clusters will arise when relying on different diagnostics, providing confusing messages to policymakers designing treatment guidelines.

## Methods

### Ethics statement

Ethical clearance for this analytical study was provided by The National Ethics Committee in Rwanda (Sep 2014, Ref No: 261/RNEC/2014).

### Detecting *Schistosoma mansoni* infections in Rwandan schoolchildren

*Schistosoma mansoni* presence-absence data was obtained from a nationwide school-based survey undertaken in Rwanda between June and mid-July 2014. Methods for school selection were as follows: a national sampling effort was carried out following the first nationwide surveys for prevalence of intestinal schistosomiasis and soil-transmitted helminthiasis (STH) in Rwandan schoolchildren, conducted between 2007 and 2008 [1]. This sampling scheme was designed to (1) provide insights into endemicity of *S*. *mansoni* across the country and (2) guide the decision of new treatment and surveillance strategies for Rwanda, in alignment with WHO guidelines [41]. The scheme took into consideration groups of sectors as administrative mapping units. Schools were chosen by selecting units likely to have similar *S*. *mansoni* transmission characteristics (based on epidemiological characteristics such as nearby perennial water bodies) and to ensure sample sizes in each mapping unit were statistically representative.

To assess possible geographical disparities in risk mapping arising from choice of diagnostic method, infection data was obtained using both the circulating cathodic antigen (CCA; detected in urine) and Kato-Katz (KK; detected in faeces) diagnostic methods. Specifically, CCA was used to survey *S*. *mansoni* infection in schoolchildren (aged 5 to 18 years) across 386 schools. A single urine sample was collected from each randomly-selected participant and tested with a point of care CCA rapid test (Rapid Medical Diagnostics, South Africa). A single-use pipette was used to add the drop of urine to the test cassette well, followed by a drop of the provided buffer. It should be noted that CCA tests returns negative, *trace*, 1+, 2++ or 3+++ readings, which are designed to give an indication of the strength of the reading. Results were read after 20 minutes and graded into one of four intensities: 0 = negative; *trace*; 1+; 2++; 3+++ using a reference image showing representative incremental readings (**Supporting Information**, S1 Fig). All CCA kits were from Rapid Medical Diagnostics Batch number 33955, ensuring we did not need to account for possible batch-to-batch variation in trace readings. We did not use a band density reader to avoid observational bias, as it would have been extremely expensive to provide such a tool to every team in the field. Moreover, we note that even though there is an mReader that Mobile Assay and SCORE, developed in 2016 for providing quantitative CCA test results, this mReader cannot distinguish between a *trace* ‘true positive’ and a *trace* ‘false positive’. Therefore, during this study and indeed to this day, the visual read of the intensity of the test band on the strip compared to the supplied control image is still considered standard practice in Rwanda.

The KK method was also used in 174 of these schools (concurrent urine and faecal samples were collected from the same participants), while a further 11 schools were surveyed using only KK (bringing the total number of schools surveyed using KK to 185). For this test, a single stool specimen was collected from each participant and duplicate thick smears were prepared for microscopic examination. Smears were assessed for presence of *S*. *mansoni* eggs by trained technicians, with each smear assessed by at least two different technicians. Quality control was performed by independent external consultants to ensure accurate diagnosis from KK microscopy. This involved random allocation of 10% of the smears for re-examination by two independent experts.

For each of the visited schools, up to 50 students per school were sampled for parasite infection. However, while the original CCA datasets included 19,371 children, only children with complete information were included in the analysis (children without age, sex, or geocoordinates recorded were excluded). As a result, the total number of children included in CCA analyses was 19,217. Following removal of children with missing data, the total number of pupils ranged from 25–50 per school for CCA sampling (mean = 49.78, sd = 1.89) and from 25–50 for KK sampling (mean = 49.59, sd = 2.05). Across the 174 schools sampled by all three methods, the number of pupils ranged from 44–50 (mean = 49.70, sd = 1.01).

### Extraction of environmental variables

Environmental measurements were extracted at the school level to be included as covariates in analyses. We extracted information for three variables likely to influence *Schistosoma* spp. survival and transmission. These included: average land surface temperature (LST), extracted from the WorldClim database (www.worldclim.org), and the Normalized Difference Vegetation and Normalized Difference Water Indices (NDVI and NDWI), both extracted from the National Oceanographic and Atmospheric Administrations’ Satellite and Information Services database (NOAASIS) (http://noaasis.noaa.gov/). LST was included because this can influence both the density of intermediate molluscan hosts and the rate of schistosomal development within the molluscan host [42]. NDVI and NDWI variables capture local variation in vegetation, moisture and the presence of water bodies, which can impact the distributions of intermediate molluscan hosts [16, 42, 43]. Environmental variables were obtained using the Google Earth Engine in ArcGIS version 10.4.0.5524 [44] at 1km x 1km grid cell resolution and were standardized to unit variance prior to analysis.

### Statistical analysis to identify infection risk factors

Our analysis assessed the presence of *S*. *mansoni* infection in 19,217 students from 386 schools who provided a urine sample for CCA assay analysis. While *trace* readings are recommended by the manufacturer and by the WHO [45] to be considered as positive infections, some programs have reported weaker *trace* readings as negative because they may not reliably confirm the presence or absence of infection [46]. For our analysis, we created two datasets for statistical analyses to account for this uncertainty when categorizing *trace* readings [47]. To do this, CCA tests were stratified into two separate presence-absence datasets, namely *CCA with trace as positive* (i.e. only those readings of 0 were considered negative, while readings of *trace*, 1+, 2+ or 3++ were considered positive) and *CCA with trace as negative* (i.e. readings of either 0 or *trace* were considered negative, while all other readings were considered positive). In addition, 9,175 students had stool specimens collected for Kato-Katz analyses, collected from 185 of the 386 schools. Data regarding school geolocation and participant demography (i.e. age and sex) was included for all observations. We stratified ages of participants into a three-level categorical variable (5 to 9 years old; 10 to 14; and 15 to 18 years old). The average age of participants included in analyses was 13.36 years. School polygon centroids were estimated from a shapefile representing Rwanda’s current administrative units (obtained from the geographic data warehouse DIVA GIS (www.diva-gis.org/Data)). Parasite infection and environmental covariate data were linked to their corresponding school centroids.

To assess evidence for spatial autocorrelation in infection probability, we fit logistic regression models (binomial errors with logit link function) using participant sex and age (categorical variables) and the scaled environmental predictors LST, NDVI and NDWI. Correlations between environmental covariates were investigated using Pearson’s correlations. Residuals were extracted and examined with semivariograms (calculated using functions in the ‘geoR’ package [48]) in R version 3.1.1 (The R foundation for statistical computing, Vienna, Austria, http://www.R-project.org). A semivariogram is a graphical representation of the spatial variation in an outcome variable; residual semivariograms represent spatial variation that is left unexplained after accounting for predictors. A semivariogram is characterised by three parameters: the partial sill, representing the spatially structured semivariance component and indicating the tendency for geographical clustering; the nugget, representing the spatially unstructured semivariance component (representing either small-scale spatial variability, measurement error or random variation); and the range, representing the pairwise distance above which two locations can be considered independent (indicative of the average size of geographical clusters). We estimated the tendency for geographical clustering within a region (i.e. proportion of variation due to spatial proximity) by dividing the partial sill by the sum of the nugget plus the partial sill [49]. Separate models were developed using KK, *CCA with trace as positive* and *CCA with trace as negative* presence-absence datasets.

### Geostatistical models

Examination of spatial autocorrelation revealed some level of residual spatial clustering for each model (see Results below). We implemented geostatistical models to account for our covariates and to simultaneously explore this autocorrelation. Bayesian logistic models with geostatistical random effects were built for CCA tests using the open software OpenBUGS [50]. We assumed the observed presence-absence vectors for each diagnostic group were random draws from an underlying infection probability according to a Bernoulli distribution. Using a logit link function, we modelled this probability as a linear regression that included an intercept, our fixed predictors and a multivariate normal geostatistical random effect capturing distances (km) between pairs of locations (using the *spatial*.*exp* function BUGS language, which is essentially a Bayesian kriging model). Note that due to a low overall prevalence in the KK dataset, we had inadequate infection data to generate precise estimates of spatial effects. We instead investigated possible spatial clustering using aggregated data by classifying locations as a binomial variable based on whether that location’s mean observed prevalence was higher or lower than the mean prevalence (1.95%) observed in the entire dataset (i.e. each survey location was categorised using 1 or 0 based on whether its prevalence was ≥ 1.95% or < 1.95%).

Geostatistical models were estimated in a Bayesian framework using Markov Chain Monte Carlo (MCMC) sampling based on the Gibbs sampler in OpenBUGS [50]. Normal priors with mean = 0 and variance = 100 (i.e. precision = 0.01) were specified for intercepts and regression coefficients. Geostatistical random effects were assumed to follow a normal distribution with mean = 0 and variance = 1/*tau*, where *tau* was drawn from a gamma distribution (shape = 0.001, scale = 0.001). For CCA models, a burn-in of 5,000 MCMC iterations was used followed by 5,000 iterations. For the KK model, a burn-in of 2,000 iterations was used followed by another 3,300 iterations. Convergence for all models was assessed visually based on inspection of posterior density and trace plots. Significance of predictor effects was inferred based on whether 95% confidence intervals of posterior estimates did not include zero.

Model predictions were used to generate representative maps of the prevalence of *S*. *mansoni* infections across Rwanda for boys aged between 5–9 years, the subgroup with the highest overall prevalence of *S*. *mansoni* infection in our dataset (though it should be noted that age was not a significant predictor of infection probability, see Results below). Predictions were made at the nodes of a 0.03 × 0.03 decimal degree grid (approximately 3km^2^). The mean and standard deviation were extracted from the posterior distributions of predicted risk. Marginal predictions of risk were calculated using the *spatial*.*unipred* command in OpenBUGS, which carries out single site predictions to yield marginal prediction intervals for each sample site.

### Estimation of the number of infections in population at risk of *Schistosoma mansoni* infections in Rwanda in 2018

To assess geographical discrepancies in *S*. *mansoni* endemicity and the estimated numbers of at-risk individuals across the three diagnostic datasets, we selected the highest risk group in our dataset, represented as boys aged between 5 to 9 years old. A raster map of the estimated total population size of this select group (estimated for the year 2018) was multiplied by the predicted prevalence of *S*. *mansoni* in the at-risk group to produce a map of the total number of infected children in each grid cell. To create the 2018 population raster, we retrieved a 2015 population density raster from the Center for International Earth Science Information Network (CIESIN) [51], which was originally estimated using National Institute of Statistics Rwanda’s Fourth Population and Housing Census 2012 data. This raster was multiplied by the reported United Nations Development Programme (UNDP) average annual rate of population change (i.e. 2.53%), which was then multiplied by the proportion of 5 to 9 year-olds (i.e. 13.5%) to produce a raster map of the estimated densities of children in this focal group in 2018. All spatial calculations and plots were conducted in ArcGIS version 10.4.0.5524 [44]. Data required to replicate analyses is included in Supporting Information, S1 Data.

## Results

### Summary statistics of *Schistosoma mansoni* prevalence in Rwandan schoolchildren

A total of 174 schools were tested for the prevalence of *S*. *mansoni* infection using all three methods, with 8,647 pupils providing both stool and urine samples. Across these samples, observed prevalence using *CCA with trace as positive* was 37.5%, 8.6% when using *CCA with trace as negative* and only 2% when using KK (Supporting Information, S1 Table). Results were broadly similar when taken across the entire dataset: only 1.95% of the 9,175 children tested with the KK method were diagnosed as infected (Table 1), while observed prevalence was 35.93% for *CCA with trace as positive* and 7.21% for *CCA with trace as negative* (N = 19,218; Table 1).

**Table 1:** Summary statistics of observed *Schistosoma mansoni* prevalence across Rwandan schools recorded using different diagnostic methods. A total of 5,547 CCA results were categorised as ‘trace’. The *CCA with trace as positive* dataset considered these as positive, while the *CCA with trace as negative* dataset considered these as negative. CCA; Circulating Cathodic Antigen.

	Kato-Katz	*CCA with trace as positive*	*CCA with trace as negative*
**Number of schools sampled**	185	386	386
**Number of pupils positive**	179	6,906	1,385
**Number of pupils negative**	8,996	12,311	17,832
**Mean prevalence (%)**	1.95%	35.93%	7.21%

**Fig 1:**
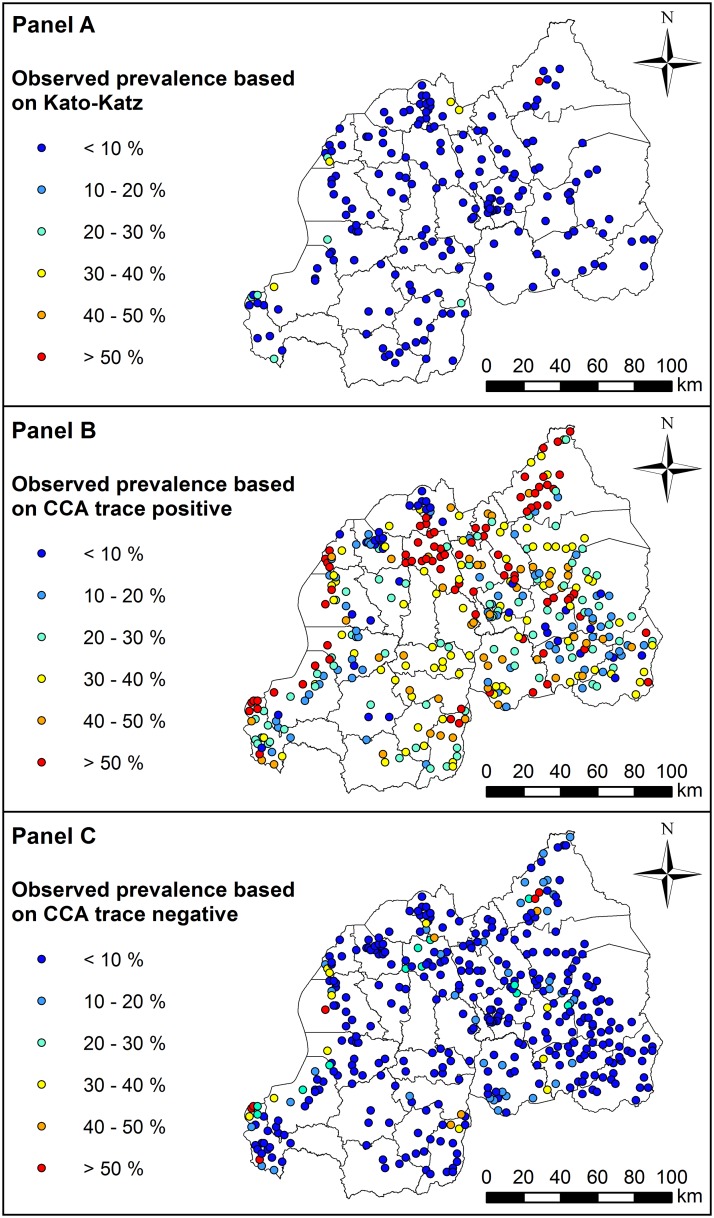
Observed prevalence of *Schistosoma mansoni* infection in Rwandan schoolchildren estimated using three different diagnostic methods: Kato-Katz (Panel A), *CCA with trace as positive* (Panel B), and *CCA with trace as negative* (Panel C). Refer to S3 Fig in Supporting Information for names of geographical districts. This figure was produced in ArcMap 10.4 (ESRI, Redlands, CA) using a shapefile representing Rwanda’s current administrative units (obtained from the geographic data warehouse DIVA GIS (www.diva-gis.org/Data)). CCA; Circulating Cathodic Antigen.

Despite these large discrepancies in diagnosed outcomes, some spatial agreement was evident across tests. Prevalence was generally highest in Northern and Western regions of the country for all three diagnostic groups (Fig 1: **Panel A to C**). However, this pattern of spatial variation was more evident for the *CCA with trace as positive* group, with many high-burden locations (i.e. with prevalence >50%) occurring within close proximity of one another in the Northern and Western regions of Rwanda (Fig 1: **Panel B**). The KK and *CCA with trace as negative* groups tended to show very low prevalence in most regions, with only a few high-prevalence locations occurring in the Northern and Western Regions (Fig 1: **Panel A and C, respectively**).

### Spatial clustering in *Schistosoma mansoni* prevalence and risk prediction

Semivariograms based on residuals from non-spatial models revealed a tendency for spatial clustering for all diagnostic groups, warranting the need for geostatistical methods (**Supporting Information**, S2 Fig). Sex of participants was an important risk factor for *S*. *mansoni* infection across all models, with males exhibiting higher risk compared to females (odds ratios of increased risk for males = 1.71, 1.08 and 1.35 for KK, *CCA with trace as positive* and *CCA with trace as negative*, respectively; Table 2). However, environmental predictors differed among methods. We found a positive association between LST and infection risk when using the KK test, whereas NDVI was positively associated with risk for both CCA groups (Table 2).

**Table 2:** Predictor associations with an individual child’s probability of being infected with *Schistosoma mansoni*, estimated using three different diagnostic methods to account for possible discrepancies in diagnostic performance and estimates of geographical endemicity. For each diagnostic method, a geostatistical model was built to estimate predictor effects while accounting for spatial autocorrelation. Presented are effect means and 95% credible intervals (in brackets). KK; Kato-Katz. CCA; Circulating Cathodic Antigen.

Variable	KK	*CCA with trace as positive*	*CCA with trace as negative*
**Age 10–14 y (versus 5–9 y)**	1.47 (-4.75, 10.15)	0.01 (-0.55, 0.57)	1.11 (-0.20, 2.73)
**Age 15–18 y (versus 5–9 y)**	0.91 (-5.35, 9.56)	-0.16 (-0.76, 0.46)	1.08 (-0.29, 2.72)
**Male (versus female)**	0.54 (0.21, 0.88)	0.08 (0.02, 0.15)	0.30 (0.18, 0.43)
**Land surface temperature**^a^	0.91 (0.14, 1.73)	0.08 (-0.03, 0.20)	0.12 (-0.06, 0.30)
**Normalized difference vegetation index**^a^	0.93 (-0.08, 2.00)	0.23 (0.08, 0.38)	0.28 (0.03, 0.54)
**Normalized difference water index**^a^	-0.36 (-1.59, 0.80)	-0.10 (-0.29, 0.08)	-0.25 (-0.56, 0.06)
**ϕ (phi: rate of decay of spatial correlation)**^b^	11.90 (4.24, 19.34)	17.36 (13.10, 19.88)	14.81 (9.06, 19.54)
**σ2 (sigma: variance of spatial random effect)**^b^	11.74 (5.93, 23.15)	1.17 (0.95, 1.46)	2.65 (2.00, 3.62)
**Tau (precision)**	0.10 (0.04, 0.17)	0.86 (0.69, 1.05)	0.39 (0.28, 0.50)
**Intercept**	-9.57 (-18.32, -2.99)	-0.80 (-1.42, -0.19)	-4.87 (-6.54, -3.48)

^a^ Variables were standardized to have mean = 0 and standard deviation = 1

^b^ Measured in decimal degrees and 3/phi determines the cluster size; one decimal degree is approximately 111km at the Equator (the size of the radii of the clusters).

**Fig 2:**
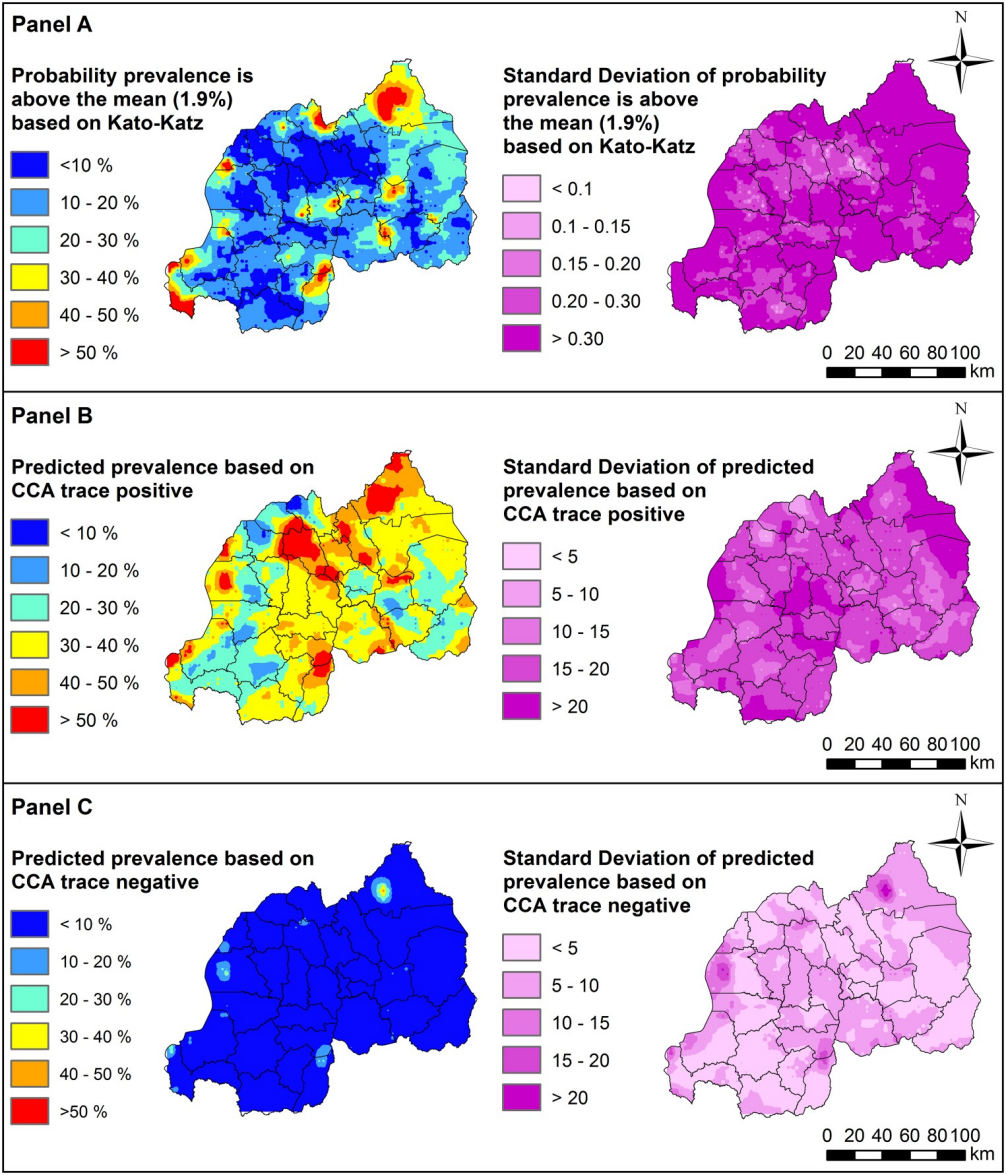
Predicted prevalence of *Schistosoma mansoni* infection in Rwandan boys aged 5–9 years, estimated using three different diagnostic methods: Kato-Katz (Panel A), *CCA with trace as positive* (Panel B), and *CCA with trace as negative* (Panel C). Predictions were generated using separate geostatistical models to account for possible discrepancies in diagnostic performance and estimates of geographical endemicity. Refer to S3 Fig in Supporting Information for names of geographical districts. This figure was produced in ArcMap 10.4 (ESRI, Redlands, CA) using a shapefile representing Rwanda’s current administrative units (obtained from the geographic data warehouse DIVA GIS (www.diva-gis.org/Data)). CCA; Circulating Cathodic Antigen.

For the KK method, regions with highest probability of harbouring above-average prevalence generally occurred in the Northern region, Nyagatare and Burera districts, the Western region such as Rusizi districts and also in inland-areas of Rwanda such as Kamonyi and Rwamagana (Fig 2: **Panel A; see** S3 Fig **in Supporting Information for locations of districts**). Predicted prevalence of *S*. *mansoni* using *CCA with trace as positive* was mostly between 30 to 40% across Rwanda, with highest prevalence (i.e. with >50% predicted prevalence) occurring in Northern districts (including Nyagatare, Gicumbi and Gakenke) as well as Southern districts such as Gisagara and Nyanza (Fig 2: **Panel B**; S3 Fig). In contrast, predicted prevalence using *CCA with trace as negative* was mostly <10% across Rwanda, with the highest predicted prevalence observed only in a small localized area in the Northern Nyagatare district (Fig 2: **Panel C**; S3 Fig). Importantly, vast areas of the country identified as high-risk when considering *trace* results as positive were estimated to have prevalence <10% when considering *trace* results as negative.

### Comparison of population densities in at-risk regions using different diagnostic tests

**Fig 3:**
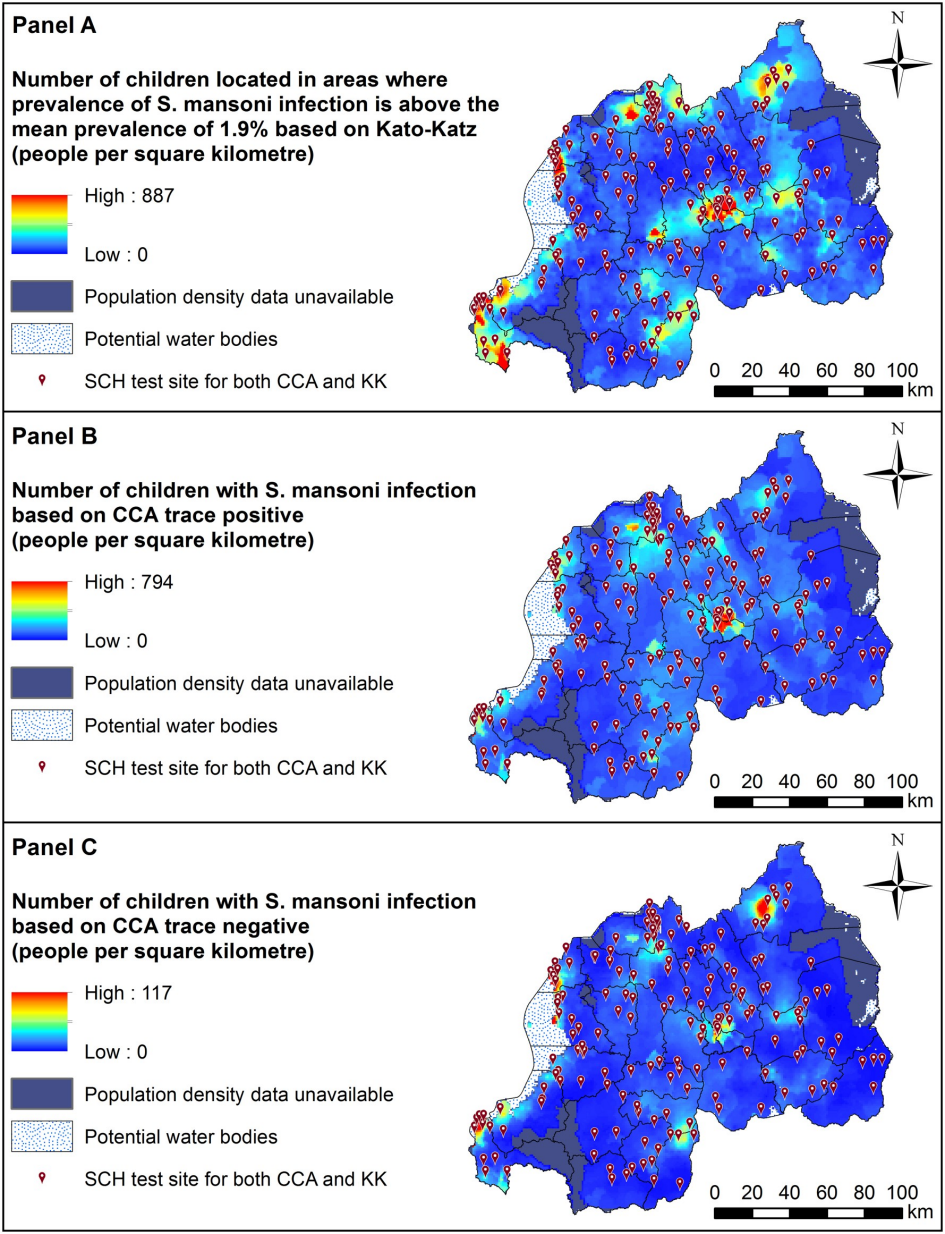
Distributions of the number of Rwandan boys aged 5–9 years (per square kilometre) estimated to be infected with *Schistosoma mansoni* based on results from three different diagnostic methods: Kato-Katz (Panel A), *CCA with trace as positive* (Panel B) and *CCA with trace as negative* (Panel C). Estimates were generated using geostatistical predictions applied to a map of Rwanda’s 2018 population. This raster was generated by multiplying National Institute of Statistics Rwanda, Fourth Population and Housing Census 2012 data [51, 52] by the reported United Nations Development Programme (UNDP) average annual rate of population change (i.e. 2.53%), which was then multiplied by the proportion of 5–9 year olds. Refer to S3 Fig in Supporting Information for names of geographical districts. This figure was produced in ArcMap 10.4 (ESRI, Redlands, CA) using a shapefile representing Rwanda’s current administrative units (obtained from the geographic data warehouse DIVA GIS (www.diva-gis.org/Data)). CCA; Circulating Cathodic Antigen. SCH; *Schistosoma* prevalence mapping unit.

Our spatially-adjusted projection maps identified areas where high densities of individuals in the focal group (boys aged 5 to 9 years) live in risk-prone environments. Projections were broadly in agreement across diagnostic tests, though it should be noted that our KK prediction map is restricted to estimating total at-risk individuals in spatial clusters with above-average predicted prevalence. Areas within and around the capital Kigali in the central area of Rwanda (i.e. the capital city Kigali, and the southwest part of Kigali including Rwezamenyo, Gitega, Kimisagara, Nyakabanda sectors) had high estimated densities of at-risk individuals across all three tests (Fig 3: **Panel A to C**; S3 Fig). In addition, target areas were also identified in localised areas around Mururu sector in Rusizi district in South-Western province, and Nkombo sector which is located around the southern end of Lake Kivu in Rusizi district.

Despite some general agreement, we identified considerable discrepancies in spatial patterns and estimated densities of at-risk children across diagnostic tests. For the *CCA with trace as positive* test, we estimated that approximately 671,856 boys aged 5 to 9 years are currently infected with *S*. *mansoni* in Rwanda (95% CI: 655,349–678,692), while only 60,453 individuals in this target group are predicted to be infected based on the *CCA with trace as negative* test (95% CI: 52,452–61,116; Table 3). While both CCA datasets identified the Gisenyi sector of the Rubavu district in the Western province as a potential target, estimated densities were much higher when using the *CCA with trace as positive* data (with up to 887 at-risk individuals per square km; compared to 117 people per square km for *CCA with trace as negative*; Fig 3: **Panel B and C**). Moreover, the KK map identified a target area around Rukomo sector in Gicumbi district (North-Eastern region) that was not identified by the CCA tests (Fig 3: **Panel A**).

**Table 3:** Predicted number of individual boys aged 5 to 9 years estimated to be harbouring *Schistosoma mansoni* infection in Rwanda in 2018 based on results from two different diagnostic methods: *CCA with trace as positive* and *CCA with trace as negative*. CCA; Circulating Cathodic Antigen.

Total population for 2015 (in Thousands)^a^	Annual population growth rate for 2015–2020 (Percentage)^a^	Percentage of individuals aged 5-9y^a^	Predicted number of individuals with *S*. *mansoni* infection in 2018	
			*CCA with as trace positive*	*CCA with as trace negative*
11629.6	2.53	13.85	671,856	60,453

^**a**^ Source: World Population Prospects 2017 Revision Population Database: Rwanda

CCA; Circulating Cathodic Antigen

## Discussion

Our study outlines important risk factors and provides national geostatistical projections of *Schistosoma mansoni* infection burden in Rwanda. Concordant spatial predictions for diagnostic test groups highlighted areas in most need of interventions. Population-dense areas in the West and around Rwanda’s capital region are projected to exhibit environmental conditions that are conducive to transmission and harbour high densities of at-risk children. Our findings therefore provide useful insights that can be leveraged to devise tailored monitoring and intervention programs. However, our assessment of multiple diagnostic procedures highlights a major issue in efforts to design intervention strategies that can reduce schistosomiasis-related morbidity. Variation in parasite detection rates, identification of different environmental risk factors and geographical discrepancies in at-risk projections suggest that considerably different inferences will be gleaned from surveys using either KK or CCA datasets [27]. This is particularly evident in Rwanda, where the low overall prevalence leads to even greater insensitivity by the KK method and is similar to a comparable country-wide survey done in Burundi [53]. We stress that data collection using multiple diagnostic sources may be necessary to generate accurate infection estimates while dealing with diagnostic test uncertainty.

### Identifying areas at greatest need of targeted *Schistosoma mansoni* mitigation: Discrepancies across Kato-Katz and circulating cathodic antigen surveys

Helminth infection is among the most common causes of anaemia and subsequent hospitalization in Rwanda [31, 54]. As one of Africa’s most densely populated countries and with an economy that relies on agriculture [32], it is unsurprising that up to 36% of schoolchildren are estimated to be infected with *S*. *mansoni*. However, infection rates are not homogenous throughout the country. Determining accurate estimates of *Schistosoma* prevalence is an important step to design mitigation programs, particularly considering that even low infection intensities can cause morbidity [3]. We provide multiple lines of evidence that different diagnostic procedures yield different inferences about *S*. *mansoni* infection rates and spatial patterns in Rwandan schoolchildren. Our prevalence estimates varied largely across procedures, with the KK method yielding far lower estimates compared to the CCA datasets. Explaining this discrepancy requires an understanding of what these tests are designed to detect. Positive detection by the KK method is limited to individuals that are infected with egg-producing female worms in sufficient numbers to consistently yield eggs in their faeces. This immediately rules out detection of some infections that are composed of male worms, of worms that have not reached sexual maturity or low numbers of worms that produce low numbers of eggs. Moreover, parasite egg counts in our study (as in most KK studies) were based on a single stool sample, which can limit the ability of technicians to identify infections [28]. Finally, other work suggests that KK detections are strongly associated with infection intensity or egg-laying rates [55]. In contrast, CCA tests are thought to provide a more unbiased estimate across heterogeneous environments [26, 56, 57]. Children who harbour pre-patent adult worms or low densities of egg-producing females are highly likely to be diagnosed as uninfected using the KK method [58], while detection of schistosome-released antigenic proteins using CCA may still be accurate [26].

Running separate analyses using multiple diagnostic procedures may not be cost-effective and clearly can lead to conflicts in resource management when attempting to reduce schistosomiasis in endemic areas. Given that CCA tests do not require a stool sample and have greater capabilities to detect low-intensity infections than KK, implementation of CCA diagnostics could be the best approach for rapid screening during ongoing monitoring programmes. However, the problem of interpretation for CCA tests still remains [56], although a recent systematic modelling paper comparing KK with CCA results from many different countries and endemic levels of infection indicates the relative comparative results of these two assays [27]. Despite the minimal training required for CCA testing, approaches to classify different trace results can be inconsistent [47, 56]. Nevertheless, as previously reported for Burundi [53], comparisons of the KK, CCA and CAA assays by latent class analysis indicate that at least half of trace results are estimated to be true positives.

Analysis of the CCA datasets in our study delivered quite different inferences. The estimated number of 5 to 9 year-old boys currently harbouring infection varied by an order of magnitude, a large difference that could be confusing to decision-makers. It should also be noted that even *CCA with trace as positive* tests are known to miss some confirmed infections [56], suggesting that our projections of burden in Rwanda could still be conservative. Because many nations where *Schistosoma* parasites are endemic do not have adequate funds for blanket treatment, this variation in prevalence estimates has important ramifications for the decision-making process. WHO guidelines are used around the world for designing mass drug administration strategies to reduce intestinal helminth infection rates in endemic nations [11, 33, 59]. Current guidelines for reducing schistosomiasis suggest that the prevalence in the at-risk school population should determine the number of interventions to use over the course of a child’s primary school years [41]. For example, areas with estimated prevalence >50% should receive treatment on an annual basis, while areas with prevalence <10% should be treat each child twice during their primary school years [41]. In light of our study, decisions about whether areas are high-risk (including many endemic clusters identified using the *CCA with trace positive analysis*) or low-risk (covering most of the country when considering the other two analyses) can lead to dramatic differences in the overall cost of treatment across diagnostic methods.

Based on our findings and on previous work suggesting a high sensitivity of CCA tests [25, 47, 57], we suggest treatment should be focused on areas that were identified by the *CCA with trace as positive* procedure as high-risk. This seems a useful approach to ensure adequate coverage of areas that likely exhibit high prevalence, high average intensity of infection and a relatively large number of infected schoolchildren. Here, our modelling identifies districts around the capital region and along the Western and Northern borders of the country (consisting of mountain highlands, the Virunga volcano range, and Lake Kivu [31]) as harbouring high *S*. *mansoni* burdens. These regions are some of the most heavily populated in the country [32, 51], and our projections indicate they contain high densities of at-risk populations. Indeed, using the less conservative *CCA with trace as positive* test, we estimate that prevalence of *S*. *mansoni* reaches >50% among schoolchildren in some of these districts. Targeting these areas will likely be the most cost-effective intervention approach for reducing prevalence and associated morbidity, as programs targeting areas with high transmission risk are expected to be more efficient reduction measures for battling schistosomiasis [10, 59].

In addition to mass drug administration, additional measures should ensure improved access to clean water and environmental measures to control snail abundances and reduce transmission [59, 60]. With malnutrition and diarrhea presenting as two of the most common causes of hospital-based child mortality in Rwanda [34], population densities and lack of adequate sanitation likely play strong roles in driving the observed spatial variation in *S*. *mansoni* infection rates. *Schistosoma* parasites maintain high transmission rates in regions where overcrowding and poor sanitation coincide [61]. For example in Rwanda’s capital city Kigali, where we identified an endemic cluster of high *S*. *mansoni* infection risk, construction of adequate sanitation facilities has not kept pace with rapid population expansion in recent years [62]. Instead, many people reside in high-density temporary slums where access to freshwater is limited. However, the issue of poor sanitation is not restricted to urbanised areas. Pit latrines, which facilitate the spread of infectious diseases through environmental contamination, are the most common toilet facilities, while defecation in fields or rivers is also commonplace [63]. Poverty contributes to these poor sanitation practices, as low-income communities are often located in marsh or swamp lands skirting urban centres in Rwanda [62]. In these areas, densities of intermediate snail hosts may be high, leading to high transmission forces.

### Risk factors for *Schistosoma mansoni* infection in Rwandan schoolchildren

Female *S*. *mansoni* worms can produce hundreds of eggs per day [64]. When sanitation rates are poor and burdens are high, ecological risk factors that influence vegetation or water properties, both of which impact parasite survival and/or infectivity, become especially important. The widespread availability of satellite imagery has played a key role in identifying ecological correlates of geographical distributions and infection rates for an incredible diversity of pathogens [29, 65–69]. For human helminth parasites, numerous geostatistical analyses have delineated spatial clusters of high infection risk, further indicating a strong role of environmental forces [29, 65, 70, 71]. In our case, we identified LST and NDVI as important predictors of *S*. *mansoni* infection probability and spatial clustering. Temperature of the land surface is a key predictor of population dynamics for intermediate snail hosts, while low temperatures can impede the development of *Schistosoma* parasites within snails [43]. Vegetation indices could reflect distributions of habitats that are suitable for snails, while both variables in tandem may influence vegetation or soil properties that determine the survival of parasite stages in human excreta (*Schistosoma* eggs that release miracidia) or the infectivity of water-borne stages that penetrate human skin (cercariae) [4, 61]. Identifying key drivers of infection risk using remotely sensed variables presents a major advantage in efforts to provide continuously updated high-resolution projection maps [66, 72–74].

### Study limitations

We provide new insights into risk factors and the spatial distribution of *S*. *mansoni* infections in Rwanda. However, our study has some weaknesses that should not be ignored. Our dataset did not consider whether infection with other intestinal parasites might have influenced risk of *S*. *mansoni* infection. Parasite co-infections are ubiquitous, and there is mounting evidence indicating that biotic parasite associations can have marked influences infection risk and/or disease progression [70, 71, 75–79]. In addition, remote-sensed variables such as those used in our study come with their own levels of uncertainty, though these are commonly ignored when producing raster maps [80, 81]. Moreover, our projection maps of at-risk population densities used population estimates from the UN population database. These estimates may not be entirely accurate, as data on the proportion of persons within our target age group overlaps a number of UN categories. Further uncertainty in our estimates could result from our approach to calculate population sizes using the UNDP average annual rate of population change. This average rate may not reflect spatio-temporal variation in population changes in Rwanda. Finally, while school-based surveys are the primary method of choice for mapping intestinal parasite infections, estimates of infections in adults would provide useful additional information to gain better insights into population-level risk factors [82].

### Conclusions

We provide high-resolution predictions of spatial heterogeneity in *S*. *mansoni* infection prevalence in Rwandan schoolchildren. Together with our identification of risk factors and data-driven projections of current burdens in at-risk populations, these results can be leveraged to make informed decisions about mass drug treatment regimes. Treatment decisions based on mapping and modelling approaches such as ours could be useful for managers deciding between sustained morbidity control or moving toward elimination [21]. Ongoing monitoring and use of continuously updated geostatistical models will be essential for designing intestinal parasite mitigation programmes and evaluating their efficacy [66, 72–74]. Nevertheless, we highlight important discrepancies in spatial disease projections that rely on different diagnostic procedures. Greater emphasis is needed to develop standardized guidelines for classifying CCA trace results, as our risk maps yielded very different conclusions about areas in need of treatment when considering traces as positive or negative. We hope that our research provides a platform to help mitigate *Schistosoma* infection burdens and associated morbidity in the tropical and subtropical regions.

## Supporting information

S1 Data Individual-level observations of infection data. (CSV)

S1 Table Associations between *Schistosoma mansoni* presence-absence results from the three diagnostic test methods. CCA; circulating cathodic antigen. The *CCA with trace as positive* dataset considered readings of ‘trace’ as positive infections, while the *CCA with trace as negative* dataset considered these as negative.
(DOCX)

S1 Fig Rapid medical diagnostic circulating cathodic antigen (CCA) test results. From the left to the right: negative (0), *trace*, positive (1+), double positive (2++), and strong positive (3+++), according to the intensity of the test line.
(DOCX)

S2 Fig Visual representations of residual spatial autocorrelation semivariograms for the Kato-Katz dataset (top panel), the CCA trace positive dataset (middle panel) and the CCA trace negative dataset (bottom panel). (DOCX)

S3 Fig Map of Rwanda’s provencial districts. (DOCX)
