# Supplementary material for: Mapping Schistosoma mansoni endemicity in Rwanda: a critical assessment of geographical disparities arising from circulating cathodic antigen versus Kato-Katz diagnostics
Source: PLoS Negl Trop Dis. 2019 Sep 30;13(9):e0007723. doi: 10.1371/journal.pntd.0007723 (PMC6786642; doi:10.1371/journal.pntd.0007723)
Supplement: S1 Table — CCA; circulating cathodic antigen. The CCA with trace as positive dataset considered readings of ‘trace’ as positive infections, while the CCA with trace as negative dataset considered these as negative. (DOCX) [file pntd.0007723.s002.docx]

**S1 Table:** Associations between *Schistosoma mansoni* presence-absence results from the three diagnostic test methods. CCA; circulating cathodic antigen. The *CCA with trace as positive* dataset considered readings of ‘trace’ as positive infections, while the *CCA with trace as negative* dataset considered these as negative.

|  |  | Scored as ‘uninfected’ | | Scored as 'infected' | |
| --- | --- | --- | --- | --- | --- |
| Kato-Katz result | N | *CCA with trace as negative* | *CCA with trace as positive* | *CCA with trace as negative* | *CCA with trace as positive* |
| uninfected | 8475 | 7858 | 5391 | 617 | 3084 |
| infected | 172 | 44 | 13 | 128 | 159 |
