# Supplementary material for: Mapping Schistosoma mansoni endemicity in Rwanda: a critical assessment of geographical disparities arising from circulating cathodic antigen versus Kato-Katz diagnostics
Source: PLoS Negl Trop Dis. 2019 Sep 30;13(9):e0007723. doi: 10.1371/journal.pntd.0007723 (PMC6786642; doi:10.1371/journal.pntd.0007723)
Supplement: S1 Fig — From the left to the right: negative (0), trace, positive (1+), double positive (2++), and strong positive (3+++), according to the intensity of the test line. (DOCX) [file pntd.0007723.s003.docx]

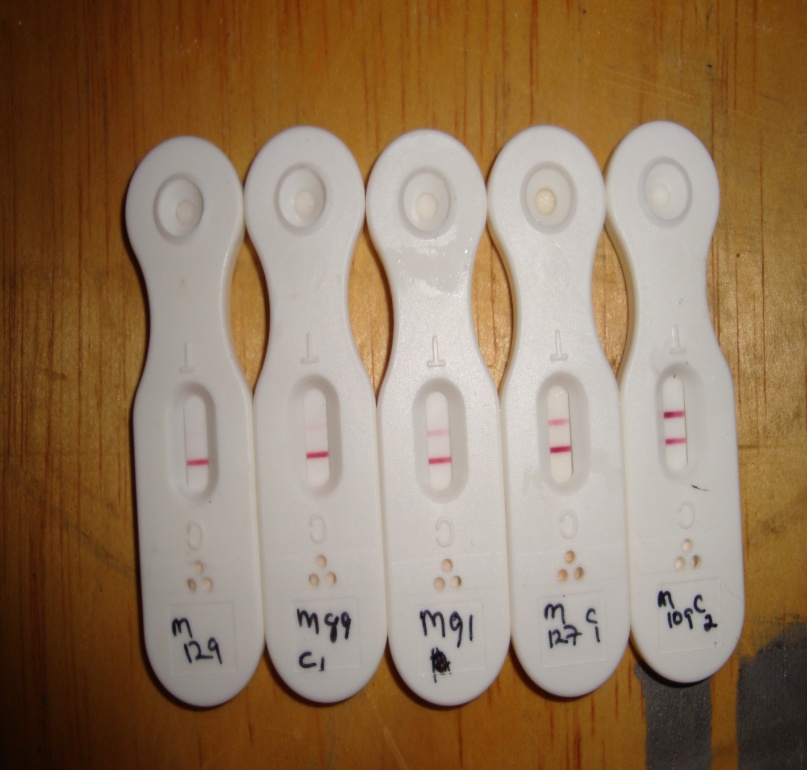


**S1 Figure:** Rapid medical Diagnostic circulating cathodic antigen (CCA) test results. From the left to the right: negative (0), *trace*, positive (1+), double positive (2++), and strong positive (3+++), according to the intensity of the test line.
