# Supplementary material for: Mapping Schistosoma mansoni endemicity in Rwanda: a critical assessment of geographical disparities arising from circulating cathodic antigen versus Kato-Katz diagnostics
Source: PLoS Negl Trop Dis. 2019 Sep 30;13(9):e0007723. doi: 10.1371/journal.pntd.0007723 (PMC6786642; doi:10.1371/journal.pntd.0007723)
Supplement: S2 Fig — (DOCX) [file pntd.0007723.s004.docx]

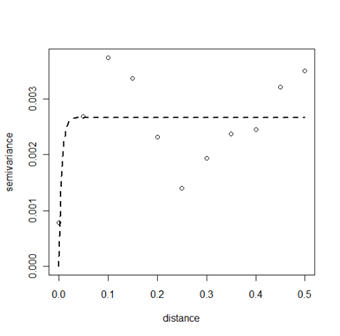

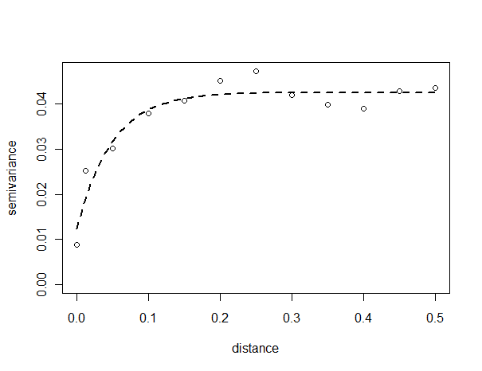

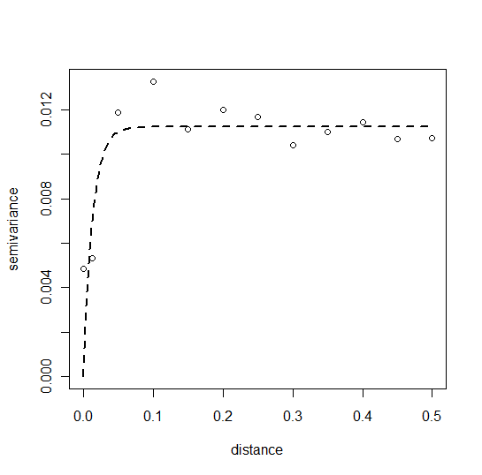


**S2 Figure:** Visual representations of residual spatial autocorrelation semivariograms for the Kato-Katz dataset (top panel), the CCA trace positive dataset (middle panel) and the CCA trace negative dataset (bottom panel).
