# Supplementary material for: Mapping Schistosoma mansoni endemicity in Rwanda: a critical assessment of geographical disparities arising from circulating cathodic antigen versus Kato-Katz diagnostics
Source: PLoS Negl Trop Dis. 2019 Sep 30;13(9):e0007723. doi: 10.1371/journal.pntd.0007723 (PMC6786642; doi:10.1371/journal.pntd.0007723)
Supplement: S3 Fig — (DOCX) [file pntd.0007723.s005.docx]

**S3 Figure:** Map of Rwanda’s provencial districts. This figure was produced in R version 3.5 using a shapefile representing Rwanda’s current administrative units (obtained from the geographic data warehouse DIVA GIS (www.diva-gis.org/Data)).
